# Dampened hippocampal oscillations and enhanced spindle activity in an asymptomatic model of developmental cortical malformations

**DOI:** 10.3389/fnsys.2014.00050

**Published:** 2014-04-14

**Authors:** Elena Cid, Daniel Gomez-Dominguez, David Martin-Lopez, Beatriz Gal, François Laurent, Jose M. Ibarz, Fiona Francis, Liset Menendez de la Prida

**Affiliations:** ^1^Laboratorio de Circuitos Neuronales, Instituto Cajal, CSICMadrid, Spain; ^2^Servicio de Neurofisiologia Clínica, Hospital General Universitario Gregorio MarañónMadrid, Spain; ^3^Universidad Europea de Madrid, Ciencias Biomédicas BásicasMadrid, Spain; ^4^Servicio de Neurobiología, Instituto Ramón y Cajal de Investigación SanitariaMadrid, Spain; ^5^Institut du Fer à MoulinParis, France; ^6^Sorbonne Universités, Université Pierre et Marie CurieParis, France; ^7^Institut National de la Santé et de la Recherche Médicale UMRS 839Paris, France

**Keywords:** oscillations, multi-site recordings, hippocampal heterotopia, epilepsy

## Abstract

Developmental cortical malformations comprise a large spectrum of histopathological brain abnormalities and syndromes. Their genetic, developmental and clinical complexity suggests they should be better understood in terms of the complementary action of independently timed perturbations (i.e., the multiple-hit hypothesis). However, understanding the underlying biological processes remains puzzling. Here we induced developmental cortical malformations in offspring, after intraventricular injection of methylazoxymethanol (MAM) *in utero* in mice. We combined extensive histological and electrophysiological studies to characterize the model. We found that MAM injections at E14 and E15 induced a range of cortical and hippocampal malformations resembling histological alterations of specific genetic mutations and transplacental mitotoxic agent injections. However, in contrast to most of these models, intraventricularly MAM-injected mice remained asymptomatic and showed no clear epilepsy-related phenotype as tested in long-term chronic recordings and with pharmacological manipulations. Instead, they exhibited a non-specific reduction of hippocampal-related brain oscillations (mostly in CA1); including theta, gamma and HFOs; and enhanced thalamocortical spindle activity during non-REM sleep. These data suggest that developmental cortical malformations do not necessarily correlate with epileptiform activity. We propose that the intraventricular *in utero* MAM approach exhibiting a range of rhythmopathies is a suitable model for multiple-hit studies of associated neurological disorders.

## Introduction

Brain structural specificity emerges during early development through elaborated genetic programs that control cell proliferation, differentiation, migration and the formation of microcircuits in close interaction with environmental factors (Rubenstein and Rakic, 2013). Malformations of cortical development result from disruptions in this intricate process and include a myriad of structural abnormalities. Malformations are grouped according to whether they are likely to reflect alterations secondary to abnormal neuronal and glial proliferation or apoptosis (e.g., focal cortical dysplasia with balloon cells), neuronal migration (lissensephaly and heterotopias) and postmigrational development (polymicrogyria and cortical dysplasia without balloon cells) (Barkovich et al., 2012). Clinically, malformations of cortical development are strongly associated with a spectrum of early-onset neurological diseases, including epilepsy, autism and intellectual disability (Norman et al., 1995; Sisodiya, 2004).

Many types of malformations of cortical development are linked with genetic mutations while others remain only clinically defined. In a recent comprehensive classification system, the emphasis is placed on a more accurate understanding of the particular disorder as a whole and not only on its genetic and clinical aspects (Barkovich et al., 2012). Advances of neuroimaging and genetic tools have resulted in an unprecedented profusion of information on gene mutations and structural abnormalities underlying a particular syndrome. It has become clear that mutations of a gene are not necessarily linked to a single syndrome and that clinical definition encompasses a variety of underlying biological processes. Mutations of different genes can similarly affect a particular cellular process or different processes downstream in their molecular pathway (e.g., LIS1 and doublecortin in microtubule function). Hence, the intricacy of these syndromes reflects the complex interaction between genetic variations, de novo mutations, early brain developmental disruptions and late secondary perturbations, i.e., the multiple-hit hypothesis. A better understanding of these processes, in terms of the pathways and pathomechanisms affecting the normal developmental program and its modulation by environmental/epigenetic factors, is a major requirement for optimal clinical classification and diagnosis. Experimental models are still required to advance in this direction.

The mitotoxic agent methylazoxymethanol (MAM) has been traditionally used to induce developmental brain dysfunction in rodents (Cattabeni and Di Luca, 1997; Colacitti et al., 1999; Battaglia et al., 2003). In this model, MAM is injected into pregnant animals; it crosses the placental barrier and produces a dose-dependent action on the embryonic brain. MAM exposure at different embryonic stages affects actively dividing neuroepithelial cells during S-phase (Matsumoto et al., 1972; Johnston and Coyle, 1979; Cattaneo et al., 1995). At E15, MAM results in a range of cortical malformations in rats, including hippocampal heterotopias and dysplasia (Zhang et al., 1995; Colacitti et al., 1999; Baraban et al., 2000). At E17, offspring display milder hippocampal heterotopias with predominant dyslamination and dysplasia in the entorhinal and prefrontal cortices (Gourevitch et al., 2004). MAM E15-treated rats exhibit enhanced excitability and are used in epilepsy research (Pitkänen et al., 2005). MAM E17-treated rats exhibit impairment of pre-pulse inhibition and behavioral abnormalities reminiscent of the positive symptoms of schizophrenia (Gourevitch et al., 2004; O'Donnell, 2011). This large spectrum of histological, behavioral and clinical effects highlights the usefulness of this model and suggests that it could be important to exert a more rigid spatiotemporal control of mitotoxic agent administration.

Here, we introduce a different model of cortical malformations in mice induced by single intra-ventricular injection of MAM to embryos *in utero*. This approach permits a better dosage, as well as temporal and spatial control as compared to the transplacental approximation. To focus on hippocampal developmental malformations potentially revelant for epilepsy research, we used embryonic stages E14–E15 to target the peak of hippocampal pyramidal cell neurogenesis (Grove and Tole, 1999; Khalaf-Nazzal and Francis, 2013). We characterized this model using combined histological and electrophysiological approaches to focus on the presence of ectopic cell groups and aberrant circuits in the neocortex and hippocampus and possible alterations of the excitatory/inhibitory balance. In addition, we compared the histo- and physio-pathological features of our model with a genetic epilepsy-prone model of hippocampal malformation, the doublecortin (Dcx) knockout mouse (Nosten-Bertrand et al., 2008; Bazelot et al., 2012).

## Materials and methods

All experimental protocols and procedures met current Spanish legislation (R.D. 1201/2005 and L.32/2007), the European Communities Council Directive of 24 November 1986 (86/609/EEC) and European Union Council Guidelines of 2003 (2003/65/CE) for animal research and they were approved by the ethics committee of the Instituto Cajal, CSIC.

### Animals

Pregnant C57BL6/6J mice were obtained from the animal facility of the Instituto Cajal. The day of detection of the vaginal plug was considered embryonic day 0 (E0). Embryos were injected intraventricularly with either MAM or saline (see below) and examined postnatally at P17 or adulthood (2–6 months). We included non-manipulated standardly bred mice as a control group. Doublecortin knockout mice (Dcx-KO) on the C57BL/6J background and their wild-type littermates were used for additional anatomical and electrophysiological studies. Dcx-KO mice (Kappeler et al., 2006) were obtained from the animal facility of the Université Pierre et Marie Curie in Paris, and the CDTA, Orleans, France, genotyped and maintained at the quarantine of the animal facility of the Instituto Cajal.

### Intraventricular *in utero* injections

E14–E15 pregnant mice were anesthetized with isoflurane and their uterine horns were exposed through the abdominal wall. Body temperature was kept constant at 37°C with a heating blanket. A volume of 0.7–3 μ l of MAM (several concentrations, see Results) or saline (NaCl 0.9%) was injected in the lateral ventricle of several embryos using a glass pipette (1.2 mm outer diameter, 0.69 mm inner diameter, Harvard Apparatus) coupled to an automatic microinjector (VisualSonics Inc, Canada) using standard approaches (Walantus et al., 2007). In initial attempts, we used ultrasound control for guiding injections (VeVo 770, VisualSonics Inc, Canada), but we found it was not required for embryos older than E14. Fast green (Sigma) was added at 0.05% to visually confirm injection. Non-injected embryos were marked with DiI (1:5 in DMSO, Invitrogen) at either the hindpaw or the thigh. The uterine horns were set back into the abdomen, which was filled with warm physiological saline, and the abdominal muscle and skin were closed with silk sutures. Immediately after surgery, pregnant mice received a subcutaneous injection of the antibiotic enrofloxacine (Baytril, 5 mg/Kg, Bayer) and an intraperitoneal injection of the analgesic meloxicam (Metacam, 0.3 mg/Kg, Boehringer Ingelheim).

Mice were examined at P3 to look for DiI marks in order to classify them as injected (MAM or saline) or non-injected animals (i.e., littermates of injected embryos that were not manipulated but shared the uterine environment). These non-injected animals were discarded for this study. We included non-manipulated mice, i.e., standardly bred mice, as a control group. Littermates of same sex were housed together at weaning. Mice were maintained under controlled conditions (temperature of 22 ± 2°C and 12:12 light–dark cycle, lights on at 7 am) with free access to food and water.

### Histological studies

Histological studies were conducted in juvenile animals (P17) and in adult mice (2–6 months) recorded electrophysiologically. Mice were perfused intracardially with 5–10 ml of PBS and 0.2% heparin followed by 10–30 ml of 4% paraformaldehyde in PBS, pH 7.4. Brains were then removed, weighted and stored in the fixative solution overnight. Coronal sections of 50 μm were cut using a vibratome. To facilitate localization and comparison with other studies we refer to comparable Bregma levels in terms of stereotaxic coordinates of the Paxinos' atlas of adult mice (in mm). One section every three, covering from 0 mm to −4 mm in anteroposterior Bregma levels, were processed for histological analyses either by toluidine blue staining (0.05%) or by immunolabeling of the neuronal protein NeuN. For NeuN immunostaining, free-floating sections were treated with 1% H_2_O_2_ for 20 min, followed by washes in 0.1 M PBS and incubation in 10% fetal bovine serum (FBS) and 0.25% Triton for 1 h. Subsequently, sections were incubated with an anti-NeuN mouse antibody (1:1000, Bachem) overnight at room temperature, washed and incubated with a biotinylated anti-mouse IgG antibody (1:200, Vector Laboratories) for 2 h. After PBS washes, the avidin-biotin-peroxidase complex (1:100, Vector Laboratories) was applied for 1 h and finally processed by 0.05% diaminobenzidine tetrahydrochloride (Sigma) and 0.01% H_2_O_2_ under stereomicroscope control. Sections were mounted on gelatin-coated slides, dehydrated and cover- slipped with DePex (Serva). Alterations in cortical and hippocampal histoarchitecture were evaluated in a stereomicroscope (S8APO, Leica) and photographed with a coupled DFC295 camera (Leica). They typically comprised subcortical and cingular heterotopias, cortical dyslamination, corpus callosal dysgenesis and hippocampal heterotopias (see below for details on quantitative analysis).

### *In vivo* electrophysiology under urethane

Adult mice (2–6 months) were anesthetized with urethane (1.7 g/kg, i.p.) in two doses of 0.85 g/kg with a 20 min interval and immediately warmed with a heating blanket to avoid hypothermia. Anesthetized animals were fastened to the stereotaxic frame and body temperature was kept constant at 37–38°C. Small holes of 1.5 mm diameter were drilled above the left hippocampus for recording spontaneous and evoked CA1 activity (anterio-posterior AP: −2.2, mediolateral ML: 1.5) and the Schaffer collateral afferent volley (AP: −1.7, ML: 1.5). An additional hole was drilled to place the ipsilateral CA3 bipolar stimulating electrode (AP: −1, ML: 0.7). Stimulation consisted of biphasic square pulses of 0.2 ms duration and amplitudes of 0.025–1 mA every 5 s. Signals were referenced against a subcutaneous Ag/AgCl wire placed in the neck. The position of the stimulation electrode was optimized to obtain a maximal population spike response at the CA1 pyramidal layer with the lowest stimulus strength at both polarities.

For recording of local field potential (LFP) activity we used linear arrays of multi-site silicon probes of 32 electrodes at 25 μm vertical spacing (NeuroNexus Tech). They were positioned to record from all strata simultaneously, from the CA1 to the dentate gyrus and CA3. Hippocampal strata were identified using information from typical LFP events including: (a) multi-unit activity to identify the CA1–CA3 stratum pyramidale and the granule cell layer of the dentate gyrus, (b) ripple events to identify CA1 cell layer; (c) sharp-wave events to define the CA1 stratum radiatum, (d) the current source density signal (second spatial derivative of LFP interspaced 25 μm) of CA1 response to ipsilateral CA3 stimulation, and (e) the spatial distribution of theta oscillations (4–12 Hz) and gamma activity (20–90 Hz), which, together with the previous information, allowed us to identify the stratum lacunosum moleculare and the molecular layer of the dentate gyrus. Layer identification was confirmed with *post-hoc* histological analysis of the probe track. Extracellular signals were preamplified (4x gain) and recorded with a 32-channel AC amplifier (Multichannel Systems, model USB-ME32-FAI-System; Germany), further amplified by 100, filtered by analog means at 1 Hz to 5 kHz, and sampled at 20 kHz/channel with 12 bit precision. Evoked field postsynaptic potentials and afferent volleys were recorded in the CA1 stratum radiatum in response to ipsilateral CA3 stimulation. Paired-pulse protocols were used to test for facilitation (using stimulation intensities of 40% of the maximal response) and inhibition (using maximal stimulation intensities) of the second response at inter-pulse intervals of 25, 50, 75, 100, 125, and 150 ms.

### Chronic video-EEG recordings

In order to look for signs of epilepsy and to further study the presence of EEG rhythmopathies in MAM-treated mice we used long-term video-EEG recording of freely moving animals. To this purpose, mice (2–6 months) were implanted with two cortical screws (1 mm diameter) above the motor cortex (AP +1.5 mm, L 1.5 mm) of both hemispheres under isofluorane anesthesia (1.5% in oxygen 30%). Temperature and physiological variables were continuously monitored with an oximeter (MouseOX, Starr Life Sci, USA). A third screw served as reference at the occipital region (AP −6 mm, ML 0 mm). All screws were placed epidurally to avoid damaging the brain. The construct was fixed to the skull with dental cement and anchored to two additional screws implanted for stability purpose. Mice were submitted to dietary care and continuous hydration over the next 1–2 days after surgery. One week after recovery and habituation to the recording cage, the EEG and video signals were monitored in sessions of 3–8 h every day, during approximately 2–3 weeks per animal. Several blocks of continuous video-EEG (up to 48 h) were also recorded over the weeks.

For video-EEG recordings, we used a wireless 1-channel EEG recording unit (Epoch, Ripple, USA). The unit consist of a transistor (R01057-6) wired to two copper electrodes connected to the implanted screws, and a receptor enclosure (50 × 27 × 31 cm) with BNC connectors to output signals in the ±5 V range with 1–500 Hz bandpass digital filter. We recorded from the right and the left electrodes in different sessions. No differences were apparent and data was pooled. The signal was subsequently amplified x5, digitalized (Digidata 1200B, Axon Instruments) and recorded at sampling rate of 2000 Hz. For simultaneous video recording, we used a webcam (Hercules HD Exchange 720p, 30 frames/sec at 320 × 240 resolution) installed at the top of the receptor cage. The video and the EEG signals were synchronized using an in-house made Arduino-based device (http://www.arduino.cc/) programmed to: (a) control a red LED that lights every 8 s (duration 1 s) on the webcam field (not visible to the animal), (b) send a synchronizing signal (square pulses of 1 s duration at 8 s interval) to the digitizer card. Synchronized video-EEG data were obtained using a dedicated graphic interface designed in Matlab (The Math Works Inc., USA).

### Systemic injection of pilocarpine and pentylenetetrazol

In order to test for pharmacological threshold for seizure induction we used intraperitoneal injection of lithium-chloride (423 mg/kg, Sigma) followed by pilocarpine hydrochloride (100 mg/kg) about 24 h after lithium injection under EEG-control in a group of implanted mice. We also used pentylenetetrazole (90 mg/kg; Sigma Aldrich) in a different cohort of animals to test for different pharmacological sensitivity. Progression to seizure activity was monitored both clinically and electrographically. We characterized different features of seizure activity to score animal response to convulsants: (a) the latency to the first EEG seizure, (b) duration of the first EEG seizure, (c) latency to the onset of *status* in pilocarpine, and (d) number of mice resistant to seizure activity.

### Data analysis

#### Histological data

Coronal sections (50 μm) stained with toluidine blue or NeuN immunolabeling were evaluated to identify and quantify cortical and hippocampal malformations. In order to standardize analysis (comparable age and brain weight) histological quantification was performed only in P17 mice. To facilitate identification of anatomical planes, all references to anteroposterior Bregma levels at P17 are inferred from equivalent sections shown in the Paxinos' atlas of adult mice and indicated in millimeters. One every three sections from both hemispheres (covering from 0 to −4 mm in anteroposterior Bregma levels) were analyzed for cingular and subcortical heterotopias. The total anteroposterior extension of each heterotopia was estimated (AP extension) and a midpoint was defined at the half distance from the first and the last sections where it appeared. When several clusters were encountered, they were considered as part of a single heterotopic complex for quantitation purposes. To evaluate potential hypomorphism of the corpus callosum, one every three sections were examined to determine the most caudal coordinate where the corpus callosum was present.

To quantify hippocampal malformations, we used sections containing the dorsal hippocampus, typically between −1 and −2.5 mm from Bregma. The volume of the dorsal hippocampus was estimated by the Cavalieri method from one every three coronal sections using the ImageJ software (NIH, USA). To characterize CA3 dyslamination we estimated the thickness of the entire stratum pyramidale, including the two layers found in MAM-treated mice. The interlayer distance was estimated from the midpoint of each layer. Measures were made at CA3b in sections at about −2 mm from Bregma. CA1 heterotopias were characterized at their midpoint section. The mediolateral and dorsoventral extensions of the heterotopia were calculated using the ImageJ software. In cases of more than one heterotopia per animal these were analyzed independently. The distance from the heterotopia to CA1 pyramidal cell layer was estimated from the centroid of the heterotopic cluster.

#### In vivo anesthetized data

Evoked postsynaptic potentials (fEPSP) and presynaptic afferent volleys (AV) were analyzed with routines written in Matlab using signals from the stratum radiatum. To build the input/output curve, we calculated the mean amplitude and slope of the fEPSP in response to 8 stimuli at growing intensities (from 25 to 900 μA) using the initial falling phase to avoid contamination with the population spike. Paired-pulse facilitation and inhibition (evaluated at the stratum pyramidale) were estimated using the ratio between the second and the first response, calculated as described for input/output curves.

To quantify potential hippocampal rhythmopathies in MAM-treated and Dcx-KO animals we performed spectral analysis of LFP signals during periods of theta (4–10 Hz) and large-irregular activity, typically present under urethane (Clement et al., 2008). For theta analysis, we selected epochs of continuous activity lasting 1–2 min. LFP signals around the stratum lacunosum moleculare were Fourier-transformed at 0.5 Hz resolution and the theta peak frequency was defined as the mean power peak of the average spectrum in the 4–10 Hz band. We chose non-overlapping data segments (1 s) with clear theta activity, as evaluated by spectral criteria. The theta frequency peak, as well as theta and gamma power, was estimated at a resolution of 1-Hz. The spectral power was given in decibels (10.log10) and calculated using a Hamming window and the Fast Fourier Transform. Theta and gamma power were defined from the spectral area in the 4–10 Hz and 30–90 Hz band, respectively. Spectral measurements were normalized by the 1/f decay at the 500–1000 Hz band.

To analyze ripple events, we chose LFP signals recorded at the stratum pyramidale of CA1 during epochs of large-irregular activity. We carefully selected sites with poor multi-unit activity to avoid high-frequency contamination. Signals were band-pass filtered between 100 and 600 Hz using a forward-backward zero-phase FIR filter of order 512 and frequency resolution of 10 Hz. To detect ripples, the band-pass filtered signal was subsequently smoothed using a Savitzky-Golay (polynomial) filter and events were detected by thresholding (>2.5 SD). Spectral analysis was performed in windows of ±200 ms centered at the detected event. Time-frequency representations were obtained by applying the multi-taper spectral estimation in sliding windows with 97.7% overlap and a frequency resolution of 10 Hz to 400 ms windows centered in ripple events. The power was estimated by averaging time-frequency spectra around the detected event. Ripple duration was evaluated by applying the Hilbert transform to the 100–600 Hz filtered signal using a threshold at 1.6 SD. The amplitude of the associated sharp-wave was estimated at the stratum radiatum.

#### Chronic video-EEG data

Continuous video-EEG data were examined by an expert clinical neurophysiologist (DML) to define well separated behavioral states: (a) active awake, typically consisting of exploratory behavior associated with dominant theta activity and (b) sleep, which was subsequently categorized as periods of REM sleep and non-REM slow-wave sleep (SWS) according to EEG information. In addition, continuous video-EEG data were examined for signs of epileptiform activities consistent of large amplitude interictal-like spikes, spike and wave components and seizure-like events. Quantitative spectral analysis was performed in the 1–50 Hz range using non-overlapping data segments of 5 s, selected from pre-defined behavioral epochs as defined above, at 1 Hz resolution. To analyze spindle activity, epochs of SWS were band-pass filtered (7–16 Hz) with a forward-backward zero-phase FIR filter of order 512, and frequency resolution of 0.5 Hz. The signal was then smoothed (Savitzky-Golay) and candidate events were detected by thresholding (>2.5 SD). Spindles were characterized by: (a) their duration, defined from the Hilbert transformation of the filtered signal at 1.6 SD threshold, (b) the spindle power in the 10–16 Hz band, estimated by averaging time-frequency spectra around each detected event, and (c) the inter-spindle interval.

#### Statistical analysis

Data are expressed as mean ± *SD*. Statistical analyses were performed using SPSS 18.0 for Windows. Normality was confirmed using the Shapiro-Wilk test. Histological comparisons were performed by One-Way ANOVA factor “MAM” followed by two-tailed Student *t*-test. Electrophysiological fEPSP responses (input/output curves and paired-pulses) were analyzed by One-Way ANOVA factor “AV amplitude” or “Stim intensity.” The spatial distribution of spectral features (power, frequency) of brain rhythms and events (theta, gamma and ripples) were analyzed with One-Way ANOVA factor “distance.” Event (ripple and spindles) features (duration, rate, spectral power and dominant frequency) were evaluated with two-tailed Student *t*-tests. In all cases we use *p* = 0.05 as significance level. Significance level was corrected for multiple comparisons whenever appropriate.

## Results

### A direct intraventricular approach to embryonic MAM injection *in utero*

**Figure 1:**
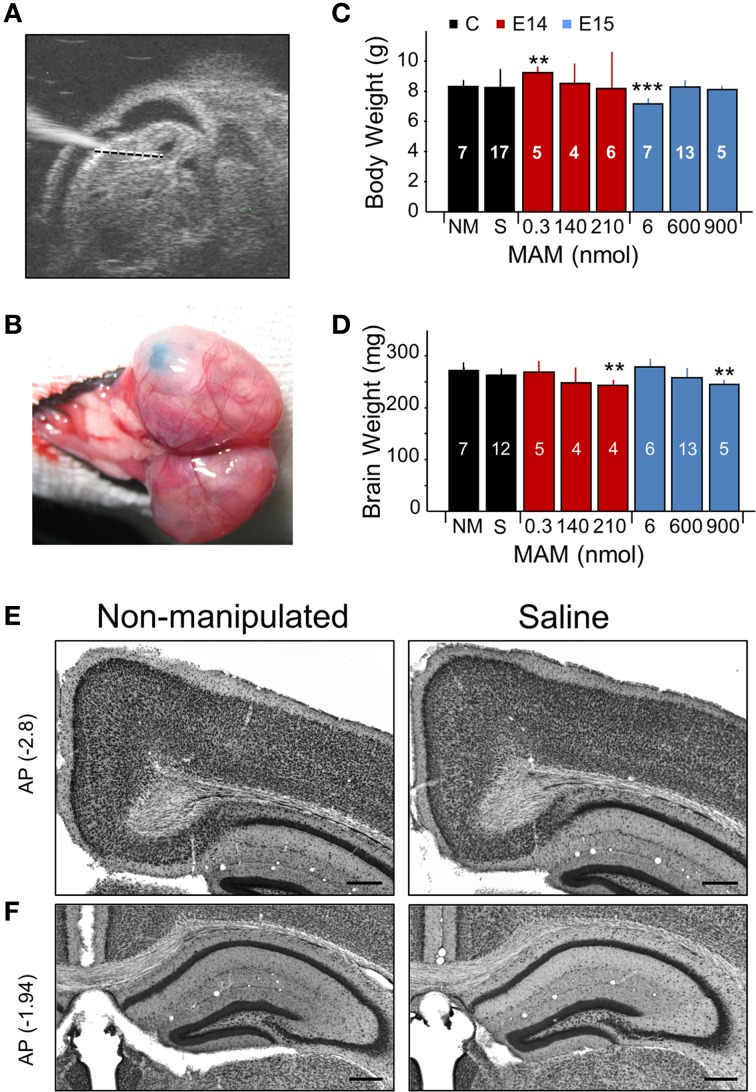
**Experimental approaches and control experiments. (A)** Different doses of methylazoxymethanol (MAM) or saline were injected intraventricularly in mouse embryos at E14 and E15 *in utero* using glass pipettes. The ultrasound image shows one embryo with the glass pipette visible on the left. **(B)** Fast green was used to visually confirm injection. **(C)** Dependence of the mouse weight at P17 on different treatments. NM, non-manipulated mice. S, saline-injected mice. Data is presented as mean ± *SD*. Number of animals are indicated at each column. **(D)** Dependence of the brain weight at P17 on different treatments, with number of animals indicated. In **(C,D)**, ^**^ (^***^) stands for *p* < 0.001 (*p* < 0.0005) in comparison with NM. **(E)** Saline-injected mice showed no major differences with non-manipulated standardly bred mice. Representative coronal sections equivalent to −2.7 to −3.0 mm posterior (AP, in mm) to Bregma were used to evaluate the cingulum. **(F)** Coronal sections equivalent to −1 to −2.5 mm from Bregma were used to evaluate the dorsal hippocampus and the callosal bundle. Scale bars correspond to 300 μm.

In order to gain a better temporal and spatial control of MAM injection compared to the transplacental approach, we established an intraventricular embryonic injection method (Figure 1A). Embryos were injected at E14 (*n* = 20) and E15 (*n* = 26) with different MAM concentrations (see below) together with 0.05% Fast Green for visual confirmation (Figure 1B). First, we noted there was a maximal MAM dose compatible with healthy pregnancy, and found that accumulated concentrations higher than 5400 nmol (corresponding to 23 mg/kg) were more likely to induce spontaneous abortion and maternal death. This is consistent with the transplacental approximation, where equivalent doses are used for intraperitoneal injections of pregnant mice (25 mg/kg). In pilot experiments we also defined the total volume per embryo and found more reliable results with 0.7 μl for E14 embryos and 1.5–3 μl for E15.

We tested a range of MAM concentrations for injections at E14 and E15 to induce developmental malformations in offspring, as examined at P17 (Figures 1C,D). Based on preliminary tests, we used concentrations of 0.3, 140, and 210 nmol for MAM injections at E14. We found more consistent malformations at 210 nmol MAM, and no obvious effect at 0.3 nmol. For injections at E15, we used 6, 600, and 900 nmol MAM and found developmental malformations in offspring for 600 and 900 nmol, and no obvious effect at 6 nmol. A major feature of developmental cortical malformations is the presence of microcephalia, which can be found both in patients and in animal models (Guerrini et al., 2008). Similar to transplacental MAM, mouse weight at P17 was rarely affected (Figure 1C) but brain weights were smaller in mice injected at E14 with 210 nmol MAM and at E15 with 900 nmol MAM vs. non-manipulated normally bred mice (*n* = 7; Figure 1D). Giving a maximal accumulated dose of 5400 nmol per pregnant mouse, we were limited to 6 embryos when MAM was injected at E15 and had no limitation (up to 25 embryos) for E14 injections.

The sham effect was evaluated by injecting E14 and E15 embryos with saline (0.9% NaCl, *n* = 20). We found no difference with non-manipulated normally bred mice either for body or brain weights (Figures 1C,D, black boxes; no significant differences) and no developmental malformations when examined histologically, either in the cingulum and neocortex (Figure 1E) or in the dorsal hippocampus (Figure 1F). We therefore pooled saline-injected and non-manipulated mice in a single control group.

### Developmental neocortical malformations in MAM-injected mice

**Figure 2:**
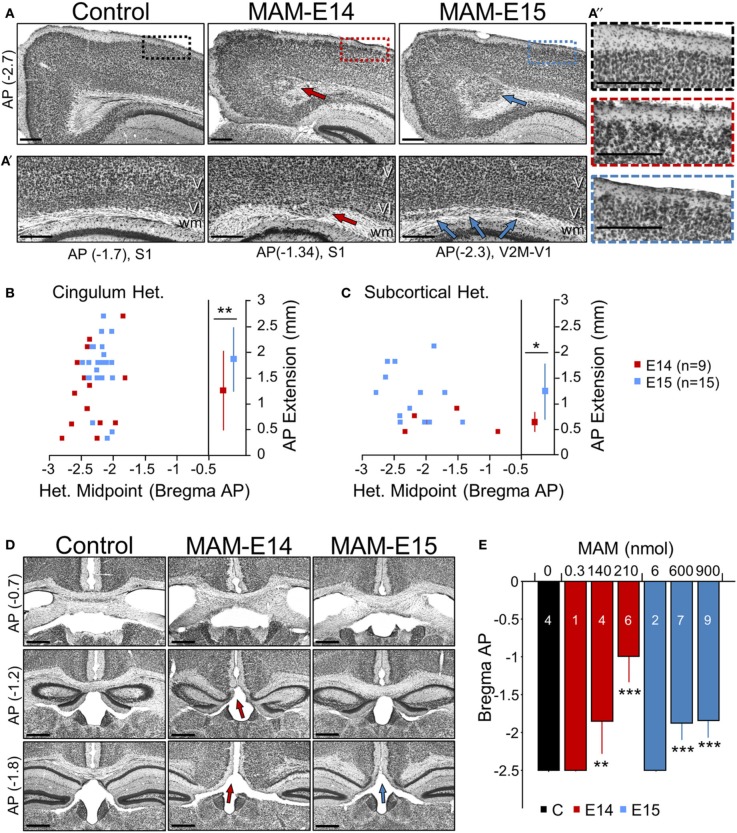
**Developmental neocortical malformations observed at P17 in MAM-injected mice. (A)** Cingular heterotopias were typically present in MAM-injected mice (arrows) at anteroposterior (AP, in mm) locations of between −3 and −2.7 mm from Bregma equivalent sections. Boxes in superficial cortical layers are expanded on the right. Scale bars correspond to 300 μm. **(A′)** Subcortical heterotopic clusters were observed at different lateral positions. Arrows indicate individual heterotopias. Scale bars correspond to 300 μm. **(A″)** Cortical dyslamination and overmigration were found to mainly affect layer II throughout the cortex. Images show an enlarged section as indicated by color boxes in **(A)**. Scale bars correspond to 300 μm. **(B)** Relationship between the cingular heterotopic midpoint and the anteroposterior (AP) extension, for mice injected at E14 (red) and E15 (blue). ^**^ stands for *p* < 0.001. **(C)** Relationship between the subcortical heterotopic midpoint and the AP extension. ^*^ stands for *p* < 0.05. **(D)** Corpus callosum hypomorphism was evaluated in coronal sections at different AP levels from Bregma (in mm). Absence of the callosal bundle was found at different levels in MAM-injected mice (arrows). Scale bars correspond to 500 μm. **(E)** Dose dependent effect of MAM on the caudal limit of the callosal bundle. Numbers indicate the animals used for quantification. ^**^ (^***^) stands for *p* < 0.001 (*p* < 0.0005) in comparison with control animals. The control group (*n* = 4) includes 2 non-manipulated and 2 saline-treated (sham) mice.

Quantitative histological analysis was performed from coronal brain sections obtained from control and MAM-injected mice at P17, but similar alterations were observed in adult animals of different ages (from 2 to 6 months). A range of developmental cortical malformations was present in both E14 and E15 MAM-injected animals. In contrast to transplacental MAM that exhibits less systematic spatial distribution of cortical heterotopias, we found precise and consistent periventricular heterotopic clusters at the cingulum (Figure 2A, red and blue arrows), similar to the cytosine arabinoside (Ara-C) model and BXD29-Tlr4lps-2J/J mutants (Ono-Yagi et al., 2000; Rosen et al., 2013). These cingular heterotopias were located under the retrosplenial cortex, typically centered between −3 and −1.5 mm posterior to Bregma, having an anteroposterior extension of about 1–2 mm (Figure 2B). They were present in 100% of E14- and E15-injected mice, although they were usually larger in the last group.

In addition, subcortical heterotopic clusters were sometimes observed at more lateral positions (under MPtA, LPtA, S1Tr, V2M, V1 regions of the cortex as abbreviated in Paxinos' atlas). They were evident either bulging into the white matter (Figure 2A′, red arrow at E14) or as well-differentiated fragmentations (Figure 2A′, blue arrows at E15). These subcortical heterotopias were more frequent and larger in E15- (73% injected mice) vs. E14-injected mice (56%) (Figure 2C). In all cases, these heterotopias were associated with cortical dyslamination and overmigration, mainly affecting layer II all along the cortex (Figure 2A″). Similar structural alterations were confirmed in adult mice (2–6 months, not shown). Therefore, intraventricular-injection of MAM at E14 and E15 resulted in cortical malformations resembling periventricular/subcortical heterotopias observed in the Ara-C and transplacental MAM model (Colacitti et al., 1998; Ono-Yagi et al., 2000; Tschuluun et al., 2011).

### Corpus callosum hypomorphism in MAM-injected mice

A number of malformations of cortical development are commonly associated with corpus callosum dysgenesis, both in humans and in animal models (Raybaud, 2010). Similarly, in MAM-injected animals we noted the absence of corpus callosum in coronal sections at caudal levels (Figure 2D), suggesting an impaired commissural genesis. This shortening of corpus callosum in the anteroposterior axis was evident in 90% of E14- and 100% of E15-injected mice, with stronger dose-dependent reduction in E14 MAM-injected animals (Figure 2E, blue bars). In adult mice (2–6 months) we confirmed similar signs of corpus callosum hypomorphism (E14: 81% mice, E15: 100% mice, not shown).

### Hippocampal heterotopias

**Figure 3:**
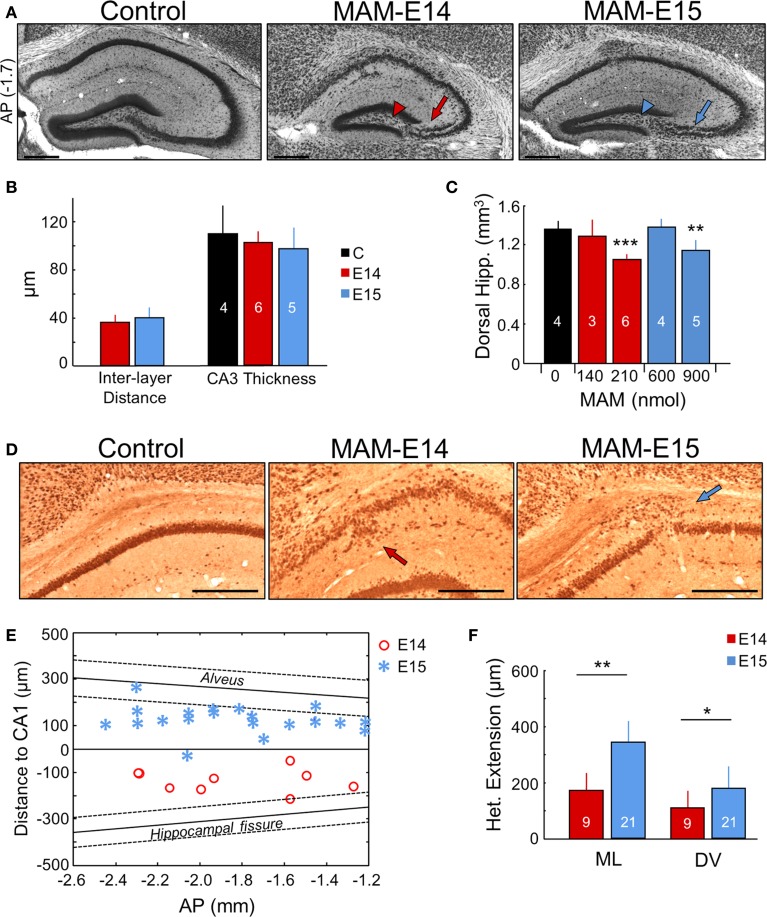
**Hippocampal heterotopias. (A)** Lamination defects, including double CA3 layer (arrows) and loss of CA3c (arrowheads) were evident at P17 and at adulthood (see Figure 6) in the dorsal hippocampus of MAM-injected mice. Scale bars correspond to 300 μm. AP, equivalent anteroposterior coordinate from Bregma (in mm). **(B)** Group data on interlayer distance and total CA3 thickness. Number of animals used for quantification is indicated. **(C)** Dose dependent effect of MAM in the volume of the dorsal hippocampus. **(D)** CA1 heterotopias were frequently found in MAM-injected mice. Images show NeuN immunostaining at P17. Scale bars correspond to 300 μm. **(E)** Position of the CA1 heterotopia in mice injected at E14 and E15 in the anteroposterior (AP) direction (x-axis) and with reference to CA1 stratum pyramidale (y-axis). In spite of variability, note different location of the center of the heterotopic clusters around the stratum pyramidale in E14 vs. E15. The anteroposterior linear trend of the alveus and the hippocampal fissure is shown for reference as mean (thick line) ± SD (discontinuous lines). **(F)** Group data of the CA1 heterotopic extension in the mediolateral (ML) and dorsoventral (DV) direction. Number of animals are indicated. The control group (*n* = 4) includes 2 non-manipulated and 2 saline-treated (sham) mice. ^*^p < 0.05, ^**^p < 0.01 and ^***^p < 0.001.

Hippocampal abnormalities are present in some types of malformations of cortical development in human (Kuchukhidze et al., 2010). In intraventricularly MAM-injected mice, we found hippocampal lamination defects and heterotopias (Figure 3A), consisting of a drastic loss of CA3c (arrowheads) and a double CA3 cell layer (arrows), similar to mice carrying mutations of Dcx (Nosten-Bertrand et al., 2008). The CA3 double layer typically appeared from −1 to −3 mm in the anteroposterior axis. The inter-layer distance in CA3 was not different between E14 and E15-injected animals (Figure 3B). Such alterations in CA3 were observed in all MAM-injected mice either at P17 or adulthood. Interestingly, segregation of CA3 stratum pyramidale into two layers did not affect the total thickness of the cell body layer (Figure 3B). Reduced hippocampal volumes were present in all MAM-injected animals, but the effect was more severe in mice injected at E14, than at E15 (Figure 3C).

A major characteristic of the transplacental MAM model is the presence of heterotopias in the pyramidal layer of CA1 (Chevassus-Au-Louis et al., 1998c; Baraban et al., 2000). In our model, these heterotopic clusters were observed in most mice injected at E15 (71% for 600 nmol MAM, and 75% for 900 nmol MAM), and were less frequent for MAM injections at E14 (50% for 210 nmol MAM, none for 140 nmol MAM) (Figure 3D). However, *in utero* MAM-injections at E14 had a more dramatic effect on CA1 dyslamination than injections at E15, leading to a more diffuse and less well organized pyramidal cell layer (Figures 3A,D). CA1 heterotopias were confirmed in adult MAM-injected mice, with similar frequency of heterotopias (E15–900 nmol: 83% mice, E14–210 nmol: 38% mice). Interestingly, we also found striking differences between mice injected at E14 and E15 with respect to the position of the CA1 heterotopia, as examined at P17 and adulthood. Mice injected at E14 exhibited larger CA1 cell layer dispersion and heterotopic clusters tended to accumulate below the pyramidal layer extending into the stratum radiatum (Figure 3E, red dots). In contrast, mice injected at E15 had a more clearly organized CA1 region and focal heterotopias were often found, but not always, over the CA1 pyramidal cell layer in the stratum oriens (Figure 3E, blue asterisks). The mean distance from the CA1 cell layer was 142 ± 48 μm in E14 and 128 ± 59 μm in E15 MAM-injected mice. In general, the extension of CA1 heterotopias was larger for E15 vs. E14, both in the mediolateral and dorsoventral directions (Figure 3F).

### MAM-injected mice exhibit higher hippocampal excitability

The heterotopias observed in CA1 and the presence of the CA3 double layer open the possibility of formation of aberrant circuits in the hippocampus of these mice, as described in the transplacental MAM approximation (Chevassus-Au-Louis et al., 1998b) and in Dcx-KO mice (Bazelot et al., 2012). While dysplastic clusters are not necessarily epileptogenic, indications suggest that it is the interaction between heterotopias and surrounding regions that potentially underlies excitability (Kobayashi et al., 2006; Ackman et al., 2009; Schwartzkroin and Wenzel, 2012). Therefore, we studied the electrophysiological properties of the dorsal hippocampus of MAM—injected mice (E14, *n* = 19) in comparison with epilepsy-prone Dcx-KO mice (*n* = 13) with similar structural alterations in CA3 (Nosten-Bertrand et al., 2008) and control animals (non-manipulated and wild-type *n* = 21; saline-injected *n* = 12). Data from wild-type littermates of Dcx-KO mice were similar to control mice data and were pooled in a control group. We focused on E14 MAM-injected animals since these exhibited more extensive hippocampal dyslamination (for E15 MAM-injected mice see below).

**Figure 4:**
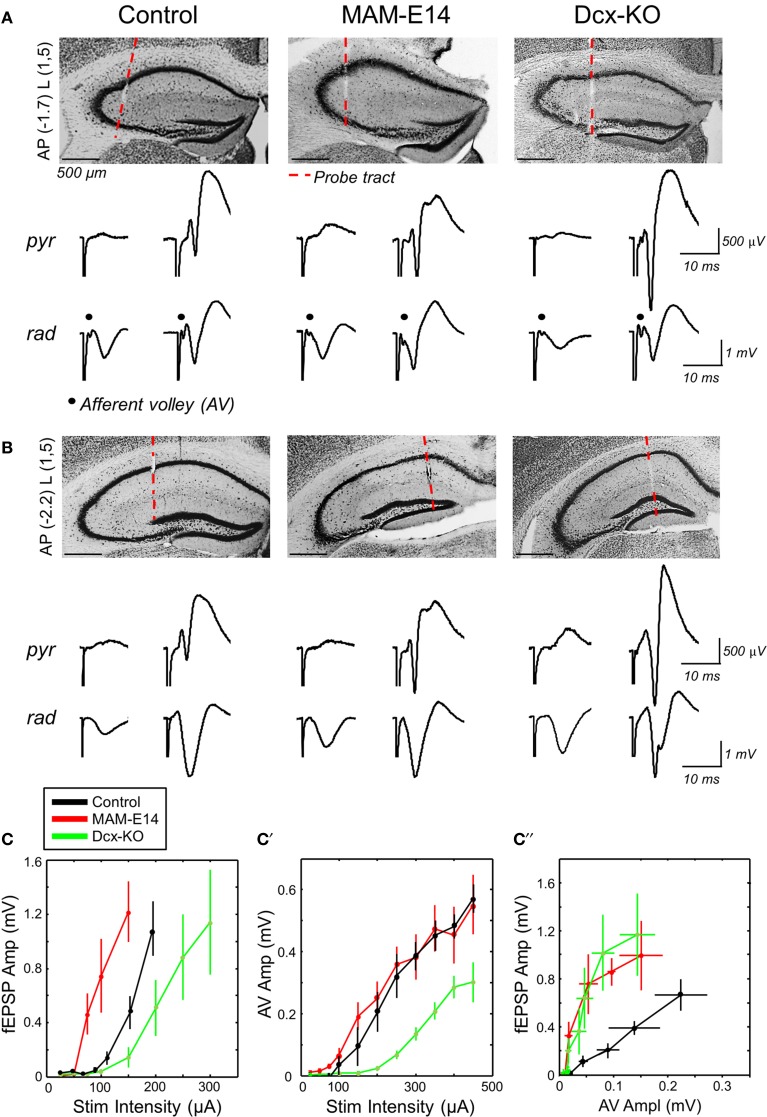
**Enhanced hippocampal excitability in MAM-injected mice. (A)** Multi-site silicon probes were used to target different layers of the dorsal hippocampus of urethane-anesthetized control and E14 MAM-injected mice. We also included doublecortin knockouts (Dcx-KO) for comparison. We recorded both the presynaptic CA3 afferent volley (AV) and the orthodromic postsynaptic response at about −1.7 mm from Bregma (AP) and 1.5 mm lateral (L). The red discontinuous line marks the probe track position. Scale bars correspond to 500 μm. We chose channels at the stratum pyramidale (pyr) and radiatum (rad), and used different stimulation intensities to evaluate responses both subthreshold (left) and suprathreshold (right) to action potential activation. **(B)** Recordings at more caudal coordinates did not show the AV response and were used to validate orthodromic responses. Scale bars correspond to 500 μm. **(C)** Relationship between the amplitude of the field excitatory potential (fEPSP) and stimulation intensity in the three experimental groups. Data from *n* = 11 control (8 non-manipulated, 3 saline injected), *n* = 6 MAM and *n* = 7 Dcx-KO. **(C′)** Relationship between the amplitude of the afferent volley (AV) and stimulation intensity. **(C″)** Input/output curves suggest enhanced excitability in MAM-treated and Dcx-KO mice, as compared to control. Data shown in **(C′,C″)** are from *n* = 6 control (4 non-manipulated, 2 saline-injected), *n* = 6 MAM and *n* = 3 Dcx-KO.

Multi-site silicon probe recordings were obtained in brains of urethane-anesthetized mice to examine input/output (IO) responses of CA1 to ipsilateral CA3 stimulation, as a proxy for hippocampal excitability. We targeted the dorsal hippocampus both at rostral (−1.7 mm from Bregma) and caudal (−2.2 mm) coordinates to examine the presynaptic CA3 afferent volley (AV, Figure 4A) and the orthodromic postsynaptic response, respectively (Figure 4B). Recording sites at the strata pyramidale and radiatum were selected to monitor both the population spike and the fEPSP.

fEPSP responses recorded at more caudal coordinates suggested stronger excitability of MAM-E14 injected mice when compared with both control and Dcx-KO (Figure 4C). Thus, the minimal current to induce a population spike was lower in MAM-E14 (193 ± 20 μA) vs. control (264 ± 23 μA, *t* = −6.33, *p* < 0.0001) and Dcx-KO mice (325 ± 61 μA, *t* = −5.06, *p* = 0.0004). These differences could be related to different excitability of the CA1 pyramidal cells themselves or to the different recruitment of presynaptic axons. We therefore examined the presynaptic AV response, which suggested poorer recruitment in Dcx-KO mice when compared with both control and MAM E14 injected animals (Figure 4C′), probably due to large dispersion of bilayer CA3 in Dcx-KO at the stimulation coordinate. As a result, IO curves from Dcx-KO and MAM E14-injected mice overlapped indicating stronger activation than in control mice (Figure 4C″). We also used paired-pulse (PP) stimulation protocols to monitor for PP-inhibition, as a measurement of GABAergic inhibitory function, and PP-facilitation, as a measure of presynaptic efficacy, and found no differences between groups (Supplementary Figure 1). In spite of these similarities, data from IO responses suggest that MAM E14-injected mice exhibit higher hippocampal excitability, similar to Dcx-KO animals which have comparable structural malformations.

### Hippocampal rhythmopathies in MAM-injected mice

**Figure 5:**
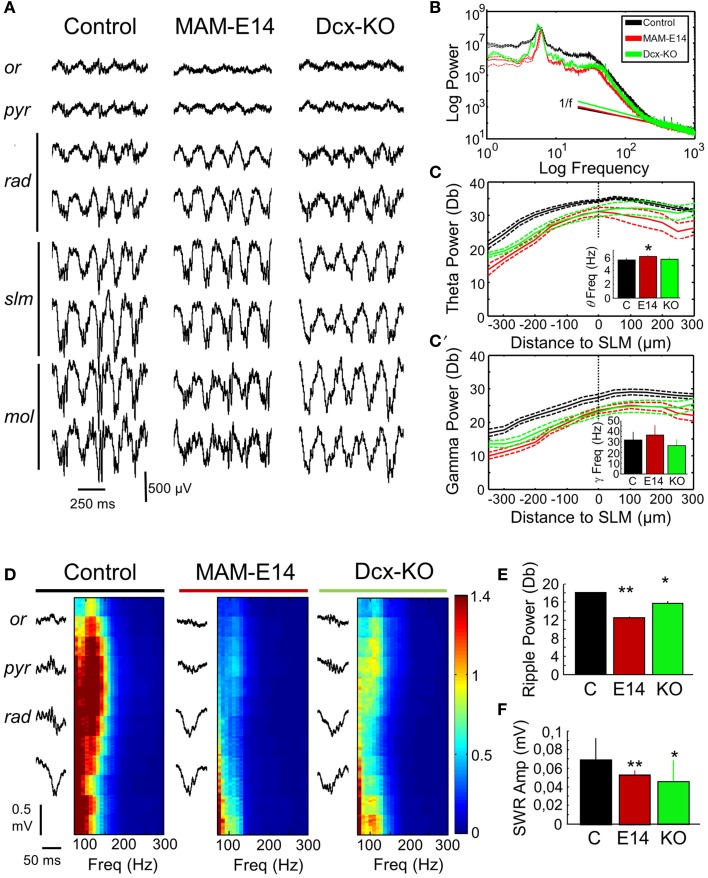
**Hippocampal rhythmopathies in MAM-injected mice. (A)** Local field potentials (LFPs) recorded from different hippocampal layers using multi-site silicon probes were examined to look at differences of theta oscillations under urethane. Note increase of theta activity at the stratum lacunosum moleculare. Or, stratum oriens; pyr, stratum pyramidale; rad, stratum radiatum; slm, stratun lacunosum moleculare; mol, stratum moleculare. **(B)** Mean power spectrum data from different groups as recorded at the slm electrode exhibiting the stronger spectral power. Discontinuous line represents 95% confidence interval. Note deviation from 1/f noise in all cases, and dominant peaks at theta (4–6 Hz) and gamma (30–40 Hz) frequencies. **(C)** Spatial distribution of the normalized (1/f corrected) theta power area (4–10 Hz) around the statrum lacunosum moleculare (taken as reference). Inset plots group differences of theta frequency peak. ^*^ stands for *p* < 0.05 in comparison against control. **(C′)** Spatial distribution of the gamma power area (30–90 Hz) around the slm (taken as reference). The inset plots group differences of gamma frequency peak. Data shown in **(B–C′)** are from *n* = 10 control (7 non-manipulated, 3 saline-injected), *n* = 8 MAM, and *n* = 5 Dcx-KO. **(D)** Potential differences of sharp-wave ripple events were evaluated using recordings from the stratum oriens, pyramidale, and radiatum. Individual events were detected (left) and their high-frequency spectrum (100–300 Hz) analyzed (right). High-frequency oscillations (ripples) at 100–150 Hz were dominant around the stratum pyramidale. Ripple power was strongly reduced in MAM-injected and Dcx-KO. **(E)** Group differences of mean ripple power as detected at the stratum pyramidale. **(F)** Group differences of sharp-wave amplitude as compared with control. Data shown in **(E,F)** are from *n* = 15 (463 events) control (299 events from 10 non-manipulated; 163 events from 5 saline-injected), *n* = 5 (123 events) MAM and *n* = 6 (217 events) Dcx-KO. ^*^ (^**^) stands for *p* < 0.05 (*p* < 0.01) in comparisons with control.

We then looked at potential alterations of hippocampal natural rhythms, recorded under urethane, i.e., theta (4–6 Hz), gamma (30–90 Hz) and high-frequency oscillations in the form of ripples (100–250 Hz). Theta oscillations were evaluated using recording sites at all hippocampal layers from CA1 stratum oriens to the molecular layer of the dentate gyrus (Figure 5A). Theta cycles in MAM-E14 and Dcx-KO exhibited the characteristic phase reversal between sites at the stratum lacunosum moleculare and the CA1 stratum pyramidale, as described in normal mice (Buzsáki et al., 2003). The power spectra of LFP signals recorded at the stratum lacunosum moleculare were similar for all groups, with a dominant peak at the theta frequency and spectral components at the gamma band (Figure 5B). However, we noted reduced theta power around the stratum lacunosum moleculare and into CA1 (defined as the area in the 4–10 Hz band) in MAM E14 [*F*_(1, 7)_ = 39.89, *p* < 0.001] but not in Dcx-KO mice [*F*_(1, 7)_ = 2.92, *p* = 0.0905] when compared to control mice (Figure 5C). Similarly, decreased gamma activity (30–90 Hz) was evident in MAM E14 [*F*_(1, 7)_ = 89.44, *p* < 0.001] and Dcx-KO mice [*F*_(1, 7)_ = 9.01, *p* = 0.0033; Figure 5C′]. These differences persisted after correction for 1/f noise as estimated from the 500–1000 Hz band (Figure 5B), to exclude potential differences in the electrode-tissue interface. However, when the theta and gamma band were normalized by the delta band, group differences vanished and no differences were confirmed in the local current source density signal (Table 1), suggesting that there was a non-specific reduction of the oscillatory power in all these bands.

**Table 1:** **Spectral features of local field potentials and current source density signals in the theta (4–10 Hz) and gamma (30–90 Hz) frequency bands**.

	**Control (10)**	**MAM E14 (9)**	***t,p* (*df* = 16)**	**Dcx KO (7)**	***t,p* (*df* = 14)**
**LOCAL FIELD POTENTIALS (LFPs)**					
Power delta (1–4 Hz)	80.43±3.93	74.09±4.82	3.1581, 0.057	77.53±2.35	1.5119, 1.528
Power theta (4–12 Hz)	91.86±2.02	87.01±3.50	**3.8285, 0.013**	88.67±3.27	2.4193, 0.297
Power gamma (20–90 Hz)	84.29±3.40	79.44±2.57	**3.3873, 0.035**	80.21±2.80	2.3381, 0.347
Theta/Delta	1.14±0.04	1.18±0.05	−1.4772, 1.579	1.14±0.02	0.0139, 9.891
Gamma/Delta	1.05±0.03	1.08±0.07	−1.1208, 2.780	1.03±0.02	0.8725, 3.977
Gamma/Theta	0.92±0.02	0.91±0.04	0.2038, 8.409	0.90±0.01	1.2655, 2.264
Frequency peak theta	5.69±0.30	6.05±0.33	−2.4517, 0.253	5.64±0.30	0.3145, 7.578
Frequency peak gamma	31.85±7.61	36.18±9.44	−1.1055, 2.844	26.48±6.11	1.3821, 1.886
Power peak theta	86.03±2.71	82.12±3.45	2.7728, 0.130	82.39±3.26	2.3435, 0.344
Power peak gamma	65.89±3.76	61.21±2.69	2.9971, 0.081	61.76±2.40	2.2362, 0.421
**CURRENT SOURCE DENSITY SIGNALS (CSDs)**					
Power delta (1–4 Hz)	65.17±3.13	71.70±9.34	−1.9875, 0.642	66.53±4.77	−0.5359, 6.003
Power theta (4–12 Hz)	69.12±4.94	71.18±5.94	−0.7976, 4.367	69.80±5.47	−0.2596, 7.989
Power gamma (20–90 Hz)	67.82±3.05	67.78±2.89	0.0227, 9.821	67.20±4.05	0.3476, 7.332
Theta/Delta	1.06±0.05	1.00±0.10	1.6182, 1.251	1.05±0.05	0.2285, 8.225
Gamma/Delta	1.04±0.04	0.96±0.11	2.1969, 0.430	1.02±0.05	1.2373, 2.363
Gamma/Theta	0.98±0.05	0.96±0.05	1.1355, 2.728	0.96±0.04	0.8062, 4.335
Frequency peak theta	5.4±0.79	6.00±0.52	−1.8973, 0.759	5.49±0.63	−0.2334, 8.187
Frequency peak gamma	50.2±0.01	47.42±8.33	1.0000, 3.321	50.20±0.01	0.0000, 10.000
Power peak theta	60.85±5.38	62.72±6.70	−0.6506, 5.244	61.27±5.68	−0.1495, 8.832
Power peak gamma	56.35±2.58	54.33±3.64	1.3521, 1.951	56.48±2.74	−0.0976, 4.235

*Data are given as mean ± SD. Number of animals are indicated in brackets. The control group includes n = 7 non-manipulated mice plus 3 saline-injected animals. All p-values are corrected for multiple comparisons (10). Comparisons with corrected p > 0.05 are shown in bold*.

We also looked at potential distortion of high-frequency oscillations (HFOs) in E14 MAM-injected mice, as HFOs are used as marker of epileptogenesis (Jefferys et al., 2012). To this purpose we focused on episodes of large-irregular activity to detect ripples (100–250 Hz) around the stratum pyramidale in association with sharp-waves at the radiatum of CA1 (Figure 5D). Sharp-wave ripple events were similarly present in all groups, in terms of rate of occurrence (control vs. MAM E14, *t* = −0.25, *p* = 0.8022; control vs. Dcx-KO, *t* = −1.38, *p* = 0.1687) and duration (Figure 5E). Similar to theta and gamma activity, the ripple power was decreased both in MAM E14 and Dcx-KO mice when compared to controls (Figure 5E). The sharp-wave amplitude was also smaller in these groups (Figure 5F).

E15 MAM-injected mice were also examined (*n* = 11, Supplementary Figure 2) and showed a similar trend, though differences were weaker than those recorded in E14 MAM-injected animals. Thus, MAM-injected mice, similar to Dcx-KO, showed hippocampal rhythmopathies reflected as a non-specific reduction of the oscillatory power of all frequency bands.

### No signs of epileptogenicity in MAM-injected mice examined under urethane

We specifically checked for signs of epilepsy in MAM-injected mice under urethane, as described in a proportion of non-anesthetized Dcx-KO animals (Nosten-Bertrand et al., 2008) and in transplacental MAM models (Chevassus-Au-Louis et al., 1998a). Surprisingly, we found no clear epileptiform events in any of the recorded animals under urethane.

**Figure 6:**
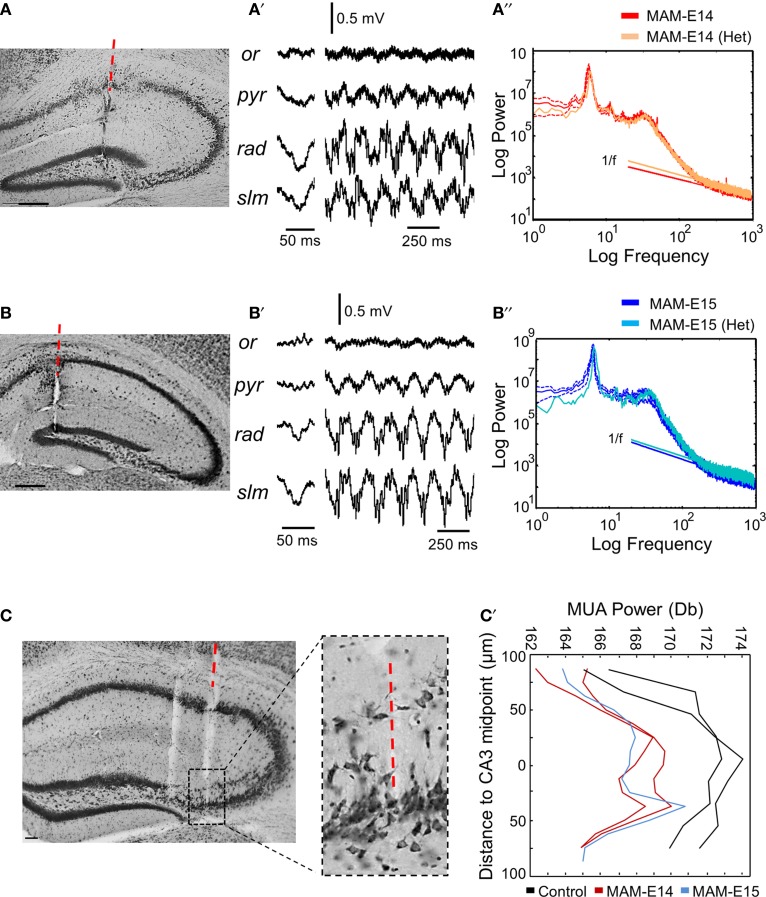
**Recordings from hippocampal heterotopias. (A)** Potential presence of epileptiform activity were evaluated in recordings restricted to heterotopic clusters. In one case of mice injected at E14, the probe track penetrated the CA1 heterotopia. Scale bar corresponds to 200 μm. **(A′)** Local field potentials (LFPs) during sharp-wave ripples and theta activity as recorded from the mouse shown in **(A)**. **(A″)** The power spectrum at the stratum lacunosum moleculare in this mouse was similar to the mean group data of mice injected at E14. **(B)** One example of mouse injected at E15, in which the probe passed through the CA1 heterotopia. Scale bar corresponds to 200 μm. **(B′)** LFPs recorded at different layers in the mouse shown in **(B)**. **(B″)** Power spectra at the stratum lacunosum moleculare in the mouse shown in **(B)** as compared with the corresponding mean group data for mice injected at E15. **(C)** Example of electrode penetration through the CA3 double layer in a MAM-injected mouse. Scale bar corresponds to 50 μm. The red discontinuous line marks the position of the probe track from where recordings were analyzed. A parallel penetration is evident at the left. The boxed region depicts an enlarged view of CA3. Scale bar corresponds to 100 μm. **(C′)** Spatial distribution of MUA power spectrum from individual cases penetrating the CA3 double layer. Data from 2 control (non-manipulated) mice are shown.

We wondered whether epileptiform activity was more likely to be restricted to heterotopic clusters. Therefore, we separately examined cases where the electrode penetration passed through the CA1 heterotopia in a mouse injected at E14 (Figure 6A) and one injected at E15 (Figure 6B). We found similar LFP profiles in these animals as those previously described for electrode penetration not targeting the heterotopia (Figures 6A′,B′), suggesting that signals are strongly dominated by volume conduction. No evidence of epileptiform events were detected in these recordings, which showed roughly similar spectral features as compared with the corresponding MAM-injected group (Figures 6A″,B″). In *n* = 3 mice (*n* = 2 MAM-E14, *n* = 1 MAM-E15), the recording probe penetrated the CA3 double layer and these cases were analyzed separately (Figure 6C). We found no evidence of epileptiform activities in these cases. Differences were only found in the spatial distribution of multi-unit activity (MUA) which gave two peaks for MAM-treated mice, consistent with the position of the double layer (Figure 6C′).

Therefore, MAM-injected mice showed no evidence of epileptogenicity under urethane anesthesia. However, since they exhibited a non-specific reduction of oscillatory power of theta, gamma and ripple activity, similar to Dcx-KO, and because a proportion of Dcx-KO mice exhibited hyperexcitability and interictal spikes when recorded chronically (Nosten-Bertrand et al., 2008), we wondered whether urethane could be masking epileptiform events in E14 MAM-injected mice.

### Chronic video-EEG recordings reveal abnormal spindle activity but not epileptiform events

Both control (*n* = 7) and E14 MAM-injected mice (*n* = 7) were implanted with epidural screws for EEG recordings coupled to video imaging for behavioral and clinical evaluation. We first focused on detecting signs of epilepsy during different behavioral phases, including active exploration, quiet wakefulness and sleep, recorded over several days.

**Figure 7:**
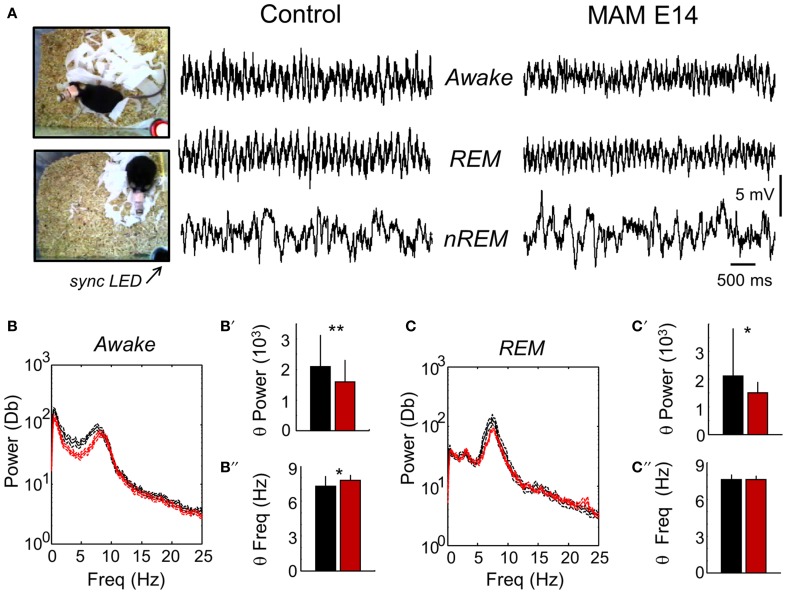
**Chronic video-EEG recordings in freely moving mice. (A)** Video-EEG recordings were obtained to evaluate epileptiform activities and to characterize the electrographic phenotype of MAM-injected animals. For these recordings we used epidural screws implanted over the motor cortex. A synchronizing LED (sync LED) was used to synchronize video images and EEG recordings, allowing for behavioral and electrographic definition of awake, REM and non-REM stages. **(B)** The mean power spectra in periods of awake activity was dominated by theta-oscillations in both groups. The discontinuous line represents the 95% confidence interval. **(B′)** Group differences of theta power as evaluated from the spectral area in the 4–10 Hz band. **(B″)** Group differences of theta frequency. Similarly as recorded under urethane, we noted an acceleration of the frequency peak. **(C)** The mean power spectra of EEG signals in periods of REM sleep was also dominated by theta-oscillations. **(C′)** Group differences of theta power in periods of REM as evaluated from the spectral area in the 4–10 Hz band. **(C″)** Group differences of theta frequency in periods of REM sleep. Data from *n* = 7 control (non-manipulated mice), *n* = 7 MAM. ^*^ (^**^) stands for *p* < 0.05 (*p* < 0.01).

We found no clear evidence of spontaneous epileptiform activity in any MAM-injected animal examined, that exhibited crudely similar EEG patterns compared with control mice (Figure 7A). During active exploration, theta oscillations dominated EEG recordings (Figure 7B), potentially reflecting volume conducted hippocampal activity (Vyazovskiy et al., 2002) and/or theta-phase modulation entrainment of neocortical cells (Sirota et al., 2008). Consistent with data under urethane anesthesia, we found lower theta power in active E14 MAM-injected mice vs. control animals (Figure 7B′). Attenuation of theta power was also detected in E14 MAM-injected mice in periods of REM sleep (Figures 7C,C′). As described for urethane, the frequency of the exploratory theta peak slightly accelerated in MAM E14 mice in comparison with controls (Figure 7B″), an effect not observed during REM (Figure 7C″). Therefore, attenuation of theta power appears to be a feature of MAM-injected mice, probably reflecting a reduction of forebrain or topographic redistribution of EEG power density as described in congenital models of callosal dysgenesis (Vyazovskiy and Tobler, 2005).

**Figure 8:**
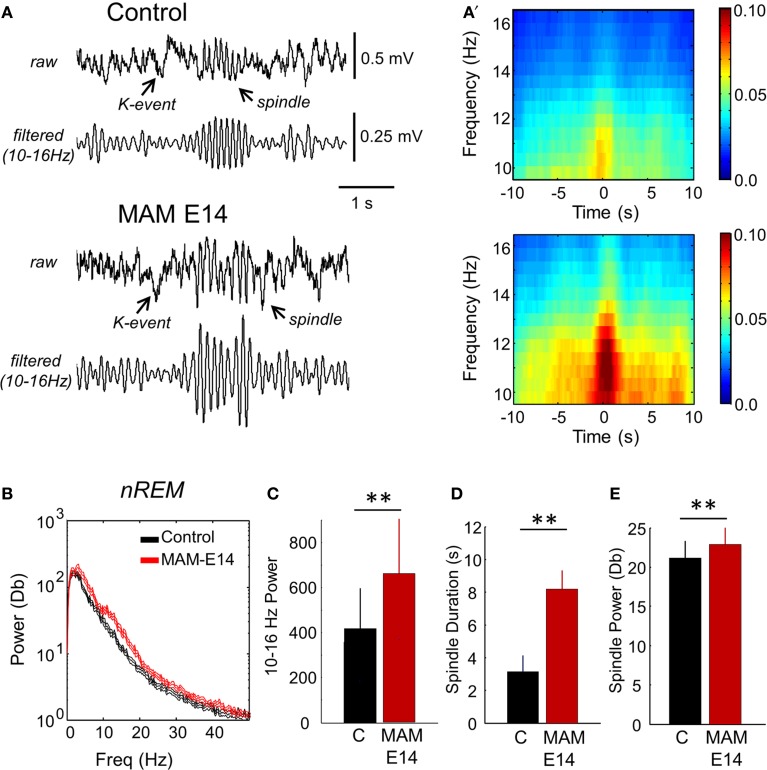
**Abnormal spindle activity during periods of non-REM sleep. (A)** Epidural EEG recordings in periods of non-REM sleep showed altered spindles in MAM-injected mice. A K-complex and the associated spindle are shown. **(A′)** Individual spindle events were detected and analyzed to highlight group differences affecting duration and spectral power in the 10–16 Hz frequency band. **(B)** Group differences of the mean power spectra in periods of non-REM sleep. The discontinuous line represents the 95% confidence interval. **(C)** Group differences of theta power as evaluated from the spectral area in the 10–13 Hz band. **(D)** Group differences of spindle duration. **(E)** Group differences of spindle power. Data from *n* = 7 control (non-manipulated mice), *n* = 7 MAM. (^**^) stands for *p* < 0.05 (*p* < 0.01).

Interestingly, we noted larger spindles in MAM E14-injected mice vs. control animals during episodes of SWS and during SWS to REM transition (Figures 8A,A′). This resulted in stronger spectral power of SWS activity in the 10–16 Hz band of the MAM E14 group (Figures 8B,C), similar to abnormal spindle activity in Williams syndrome and in a case of double cortex syndrome (Sforza et al., 2010; Bódizs et al., 2012). Spindles in MAM-injected mice were not evident during REM or wakefulness, and during sleep they were typically associated to K-complexes (but not always). Detection and spectral analysis of individual spindles (Figure 8A′), showed they lasted longer (Figure 8D) and had stronger spectral power (Figure 8E) in E14 MAM-injected vs. the control group. Our surface macroelectrode data did not allow us to reliably explore potential contribution from low-voltage and high-voltage spindles (Johnson et al., 2010), and this is open to further research. Spindle rate was not different between groups (not shown). Abnormal spindle activity could be potentially associated with other behavioral abnormalities in MAM models of psychiatric disorders (Phillips et al., 2012).

### Convulsant agent administration discards epilepsy-related phenotypes

Since thalamocortical spindle activity has been associated with some forms of generalized epilepsy (Gloor and Fariello, 1988; Danober et al., 1998), we tested whether MAM-injected animals were more sensitive to convulsants than control mice. We chose to induce generalized-like seizures using pentylenetetrazol in a group of implanted animals (*n* = 7 MAM E14, *n* = 6 control) and temporolimbic-like seizures using pilocarpine injections (*n* = 7 MAM E14, *n* = 5 control) under EEG control. We found no difference in the latency to *status* induced with lithium-pilocarpine, neither in the time to the first electrographic seizures with pentylenetetrazol (Table 2), in contrast to previous data from transplacental MAM models and Dcx-KO mice (Chevassus-Au-Louis et al., 1998a; Nosten-Bertrand et al., 2008). Of note, the time to the first pilocarpine-induced seizure was longer in MAM-injected mice and they exhibited fewer seizures before the *status* onset as compared with control (Table 2), suggesting that once an hyperexcitable state is reached they quickly progress into status. This might be related with higher excitability of the model, as confirmed with hippocampal I/O curves (Figure 4).

**Table 2:** **Characterization of seizure progression and susceptibility to pilocarpine and pentylenetetrazol systemic injections**.

	**E14 MAM-injected**	**Control**
**LITHIUM-PILOCARPINE**		
Time to first electrographic seizure (sec)	1045±469	640±117^*^
Total number of seizures before *status*	0.8±0.8	2±0.6^*^
*Status* onset (sec)	1089±575	1533±572
% of resistant mice	1/7	0/6
**PENTYLENETETRAZOL**		
Time to first electrographic seizure (sec)	177±87	232±185
First seizure duration (sec)	21±5	19±2
% of resistant mice	1/7	1/5

*Data are represented as mean ± SD*.

* *stands for p < 0.05 for two-tailed t-test statistical comparisons between groups. The control group include only non-manipulated mice*.

In view of these combined electrophysiological data we conclude that intraventricularly MAM-injected mice were not obviously prone to generalized and temporolimbic epilepsy, in spite of a range of cortical and developmental hippocampal malformations and structural similarities with other epilepsy-prone models. Instead they exhibited a range of rhythmopathies affecting thalamocortical and hippocampal activities.

## Discussion

We generated a model of cortical dysplasia in mice induced by intraventricular MAM injection *in utero* aimed to gain better spatiotemporal control of mitotoxic agents. Mice injected at E14 and E15 exhibited a range of cortical and hippocampal malformations that recapitulate different histological phenotypes of specific genetic mutations and transplacental injections. These include nodular heterotopias in the cingulum, similar to the transplacental Ara-C and MAM model (Ono-Yagi et al., 2000; Tschuluun et al., 2011); corpus callosum dysgenesis similar to congenitally affected recombinant inbred lines (Schimanski et al., 2002) and hippocampal malformations also present in Dcx- knockouts and transplacental MAM injection (Ackman et al., 2009). In contrast to most of these models, intraventricularly MAM-injected mice were not more prone to epilepsy, in spite of enhanced hippocampal excitability and a range of structural malformations. Instead they showed non-specific reduction of the spectral power of hippocampal-related brain oscillations, including theta, gamma and HFOs; and longer and enhanced thalamocortical spindle activity during non-REM sleep stages. We conclude that intraventricular MAM *in utero* is a histological asymptomatic model of cortical malformations exhibiting a range of rhythmopathies, which can be further exploited to look at the complex interaction between early disruptions of brain development and vulnerability to later hits.

Malformations of cortical development are associated with a variety of syndromes, including epilepsy, intellectual disabilities and autism spectrum disorders. While many types of malformations are related to gene mutations, others are only defined based on clinical criteria (Barkovich et al., 2012). However, there is no clear separation between these categories. For instance, periventricular heterotopias can be defined clinically with unknown causes, or genetically with autosomal dominant inheritance of different genes (Barkovich et al., 2012). Mutations of the alpha tubulin 1A, LIS1 and doublecortin genes are all associated with classical forms of lissencephaly (Gleeson et al., 1998; Keays et al., 2007; Wynshaw-Boris, 2007), with LIS1 and DCX mutations causing different malformation gradients along the fronto-occipital axis (Pilz et al., 1999). DCX mutations may also course with intellectual disability and/or epilepsy giving raise to subcortical band heterotopia in females and also occasionally in males (Guerrini et al., 2003). Mutations affecting microtubule proteins, such as alpha tubulin 1A, are also associated with cortical dysgenesis varying from mild to severe pachygyria with or without corpus callosum dysgenesis and cerebellar hypoplasia (Kumar et al., 2010). Neurological disorders associated with this mutation vary substantially, from moderate to severe intellectual disability with or without refractory epilepsy (Bahi-Buisson et al., 2008). Possibly, LIS1, DCX, and alpha tubulin 1A mutations have different phenotypes because the ultimate phenotype depends upon the precise mutation, the effects of the mutation on the structure of the molecular product of the gene and the effects of the altered structure of the molecular product on the molecular function in the molecular pathway under epigenetic influences (Dempster et al., 2011; Feil and Fraga, 2012; Bahi-Buisson et al., 2013). Furthermore, single-nucleotic variations, somatic mutations and constitutional aneuploidy in the normal human brain may also contribute to heterogeneous phenotypes and functional diversity (Rehen et al., 2005; Poduri et al., 2013). This reflects the intrinsic complexity of neurodevelopmental disorders.

Our data suggest that cortical developmental malformations do not necessarily correlate with major brain malfunction. This would suggest that imaging studies (PET, fMRI) in people suffering from epilepsy, excluding idiopathic epilepsies, may not be fully informative in some cases (Duncan, 1997; King et al., 1998). However, some experimental studies (Manent et al., 2009; Lapray et al., 2010) demonstrated good relationship between the size and location of the malformation with the functional deficits. This is consistent with the fact that some but not all forms of focal cortical dysplasia represent neuropathological entities with specific neuroimaging and electrographic features (Tassi et al., 2002; Urbach et al., 2002). Possibly, the true relationship between anatomical alterations and brain function is hidden in the asymptomatic human population that has not reached the diagnostic threshold of a developmental brain disorder.

The absence of any obvious seizure-related phenotype in MAM-injected mice, neither spontaneously nor pharmacologically-induced, appears at odds with previous models. Decreased seizure thresholds have been reported for transplacental MAM models using a variety of convulsants to trigger generalized, limbic-like or secondarily generalized seizures (Baraban and Schwartzkroin, 1996; Germano and Sperber, 1997; Chevassus-Au-Louis et al., 1998a). Most of these experiments have been carried on rats, and potential species and genetic background factors could explain differences (Xu et al., 2004). Dcx-KO mice carrying the CA3-double layer and enhanced excitability can express both spontaneous seizures and a higher susceptibility to kainate- and pentylenetetrazol-induced seizures (Nosten-Bertrand et al., 2008). This appears consistent with the increased hippocampal excitability in these models, as tested both *in vitro* (Avoli et al., 1999; Colacitti et al., 1999; Baraban et al., 2000; Bazelot et al., 2012) and *in vivo* (Figure 4). Also, Lapray et al. (2010) using an acute Dcx RNA interference model showed a correlation between the size of neocortical heterotopias and seizure severity. However, several other models show dissociation between the presence of heterotopias and the epileptic phenotype, i.e., the Eker rat model of tuberous sclerosis showed no spontaneous seizure activity and a similar seizure threshold compared to control animals (Wenzel et al., 2004; Tschuluunm et al., 2007). Therefore, developmental dysplastic lesions appear to be not necessarily epileptogenic. Possibly, dampened hippocampal rhythms and abnormal thalamocortical spindles recorded in our model could be related with cognitive impairment associated with rhythmopathies in neurodevelopmental disorders and epilepsy (Uhlhaas and Singer, 2012; Inostroza et al., 2013).

The debate is open for alternative explanations and concepts. One possibility is that it is the interaction between heterotopias and the surrounding circuits that controls epileptogenesis (Chevassus-Au-Louis et al., 1998b; Kitaura et al., 2012; Schwartzkroin and Wenzel, 2012). Concerning Dcx RNAi induced neocortical heterotopias, Ackman et al showed synchronization between the heterotopia and the overlying hyperexcitable cortex, similar to data in subcortical band patients by Kobayashi et al. (2006). This suggests that abnormal connectivity in terms of excitatory circuits and GABAergic inhibitory control could potentially underlie hyperexcitability in epileptogenic regions (Roper et al., 1999; Sarkisian et al., 2001; Menendez de la Prida et al., 2002; Alonso-Nanclares et al., 2005; Calcagnotto et al., 2005; Poluch et al., 2008). Hence, understanding altered connectivity and transmission within dysplastic regions and with other brain areas, may be critical to understand resistance to epilepsy given the role of interneurons in controlling the expression of epileptic phenotypes (Lodato et al., 2011; Powell, 2013). Possibly, interfering roles of independent epileptogenic circuitries could also account for this, as in the case of thalamocortical and corticolimbic circuits which are proposed to mutually inhibit each other (Eşkazan et al., 2002; Gurbanova et al., 2008). We indeed found here longer and stronger thalamocortical spindle activity in MAM-injected mice (Figure 8), which could potentially modulate hippocampal hyperexcitability.

Alternatively, it is possible that the diffuseness of cortical malformations disrupts the natural propagation pathways of neuronal activity therefore segregating local enhanced excitability to dysplastic regions. For instance, many cortical malformations are typically associated with corpus callosum dysgenesis (Raybaud, 2010), as we also found in intraventricularly MAM-injected mice (Figure 2). This, together with haphazard dyslamination of neocortical layer II, could potentially impair activity spread and reverberation in a critical mass of affected circuitries. Interestingly, partial agenesis of the corpus callosum and the position of heterotopia appear to modulate seizure susceptibility (Gabel et al., 2013). Notably Dcx KO mice on the C57BL/6J background studied here have an intact corpus callosum (Kappeler et al., 2007).

The expression of a particular clinical phenotype could strongly depend on co-existing pathologies in interaction with environmental factors, as suggested from case reports of polymicrogyria (Barkovich et al., 1995) and of focal dysplasia and hippocampal sclerosis with a history of febrile seizures (Lévesque et al., 1991; Fauser et al., 2006; Gibbs et al., 2011). Similarly, tuberous sclerosis is associated with very high risk of autism spectrum disorders when tubers in the temporal lobe are associated with early-onset seizure activity (Bolton et al., 2002; Numis et al., 2011). In a postnatal freeze lesion model of polymicrogyria, epileptiform activity is recorded adjacent to the lesion but not in the microgyrus itself (Jacobs et al., 1996). This model shows surprisingly widespread cortical modifications in receptor density (Zilles et al., 1998) and GABA receptor subunits (Redecker et al., 2000), although the anatomical lesion (microgyrus) is very local. Interestingly, freeze lesions appear to become epileptogenic when they are executed prenatally *in utero* (Takase et al., 2008) suggesting that the timing of brain malformations is a critical factor.

Different animal models of malformations of cortical development are thus key tools to better understand these associations. Both genetic and pharmacological approaches have been used extensively to determine which proteins and pathways play roles in the intricate processes of brain development. Yet, while our understanding of the normal developmental program is more sophisticated (Rubenstein and Rakic, 2013), we still lack a holistic view of the diseased developmental brain. What determines the expression of a particular neurological feature in association with a particular form of cortical malformation? What is the relative contribution of genetic and environmental factors? Are there common signaling pathways underlying different types of cortical malformations? These questions incite the identification of new models for more accurate clinical definitions. To account for the genetic, developmental and clinical complexity of cortical malformations, models should probably allow an independent early brain disruption with a late second hit. This two-hit approach is now being considered to better model complex diseases like cancer and schizophrenia, which are linked to several genetic and environmental factors. The intraventricular MAM model of cortical dysplasia with hippocampal and thalamocortical rhythmopathies could be combined with genetic and seizure susceptibility models to study associated neurological disorders. This could include using intraventricular MAM injection in modified genetic backgrounds (e.g., upstream to the mammalian target of rapamycin mTOR cascade; Baybis et al., 2004; Orlova et al., 2010) and the manipulation of environmental factors (stress, seizure induction, hyperthermia, etc.) to causally test and to dissect out critical signaling pathways and syndromes underlying the wide spectrum of developmental cortical malformations.

### Conflict of interest statement

The authors declare that the research was conducted in the absence of any commercial or financial relationships that could be construed as a potential conflict of interest.

## Supplementary material

Supplementary Figure 1 **Paired-pulse inhibition and facilitation. (A)** (Left) Paired-pulse inhibition (using maximal stimulation intensities) of the second response at inter-pulse intervals of 25, 50, 75, 100, 125, and 150 ms was used to probe for inhibitory circuits in different groups of animals, including doublecortin knockouts (Dcx-KO). (Right), No differences were evident between groups. Data from *n* = 9 control (non-manipulated), *n* = 6 MAM and *n* = 7 Dcx-KO. Saline-injected mice were similar to control. **(B)** (Left) Paired-pulse facilitation (using stimulation intensities of 40% of the maximal response) of the second response was used to probe for differences on presynaptic release. (Right), No clear differences emerged between groups. Data from *n* = 7 control (non-manipulated), *n* = 8 MAM and *n* = 8 Dcx-KO. Saline-injected mice were similar to control.

Supplementary Figure 2 **Electrophysiological recordings in E15 MAM-injected mice under urethane. (A)** Relationship between the amplitude of the fEPSP and stimulation intensity. Data from *n* = 11 control (non-manipulated), *n* = 5 MAM, and *n* = 7 Dcx-KO. **(A′)** Relationship between the amplitude of the afferent volley (AV) and stimulation intensity. **(A″)** Input/output curves suggesting slightly higher excitability in E15 MAM-treated mice as compared to control. Data in **(A′,A″)** are from *n* = 5 control (non-manipulated), *n* = 3 MAM, and *n* = 3 Dcx-KO. Saline-injected (sham) mice were similar to control. **(B)** Group data on paired-pulse facilitation (*n* = 7 control, *n* = 5 MAM, and *n* = 8 Dcx-KO). **(B′)** Group data on paired-pulse inhibition. (*n* = 9 control non-manipulated mice, *n* = 4 MAM, and *n* = 7 Dcx-KO). No differences were found between groups. Saline-injected (sham) mice were similar to control. **(C)** Mean power spectrum data from control (black, *n* = 10 non-manipulated mice) and E15 MAM-injected mice (blue, *n* = 5) as recorded at the slm electrode. Discontinuous line represents 95% confidence interval. Note deviation from 1/f noise in all cases, and dominant peaks at theta (4–6 Hz) and gamma (30–40 Hz) frequency. **(C′)** Spatial distribution of the normalized (1/f corrected) theta power area (4–10 Hz) around the stratum lacunosum moleculare (taken as reference). There is statistical difference between groups especially at CA1 [*F*_(1, 7)_= 32.03; *p* < 0.001]. Inset plots group data on theta frequency peak showed no difference at the stratum lacunosum moleculare. **(C″)** Spatial distribution of the gamma power area (30–90 Hz) around the slm channel (taken as reference) showed differences between groups [*F*_(1, 7)_ = 44.87, *p* < 0.001]. The inset plots group differences of gamma frequency peak. **(D)** Potential differences of sharp-wave ripple events in control and E15 MAM-injected mice. High-frequency oscillations (ripples) at 100–150 Hz were maximal around the stratum pyramidale. **(D′)** Similar to E14 injected mice, ripple power was strongly reduced in E15 MAM-injected animals. **(D″)** No group differences were found in ripple duration. Data from *n* = 10 (463 events) control (non-manipulated mice), *n* = 5 (224 events) MAM, and *n* = 5 (217 events) Dcx-KO.
