# Supplementary figures and images for: Dampened hippocampal oscillations and enhanced spindle activity in an asymptomatic model of developmental cortical malformations
Source: Front Syst Neurosci. 2014 Apr 14;8:50. doi: 10.3389/fnsys.2014.00050 (PMC3995045; doi:10.3389/fnsys.2014.00050)

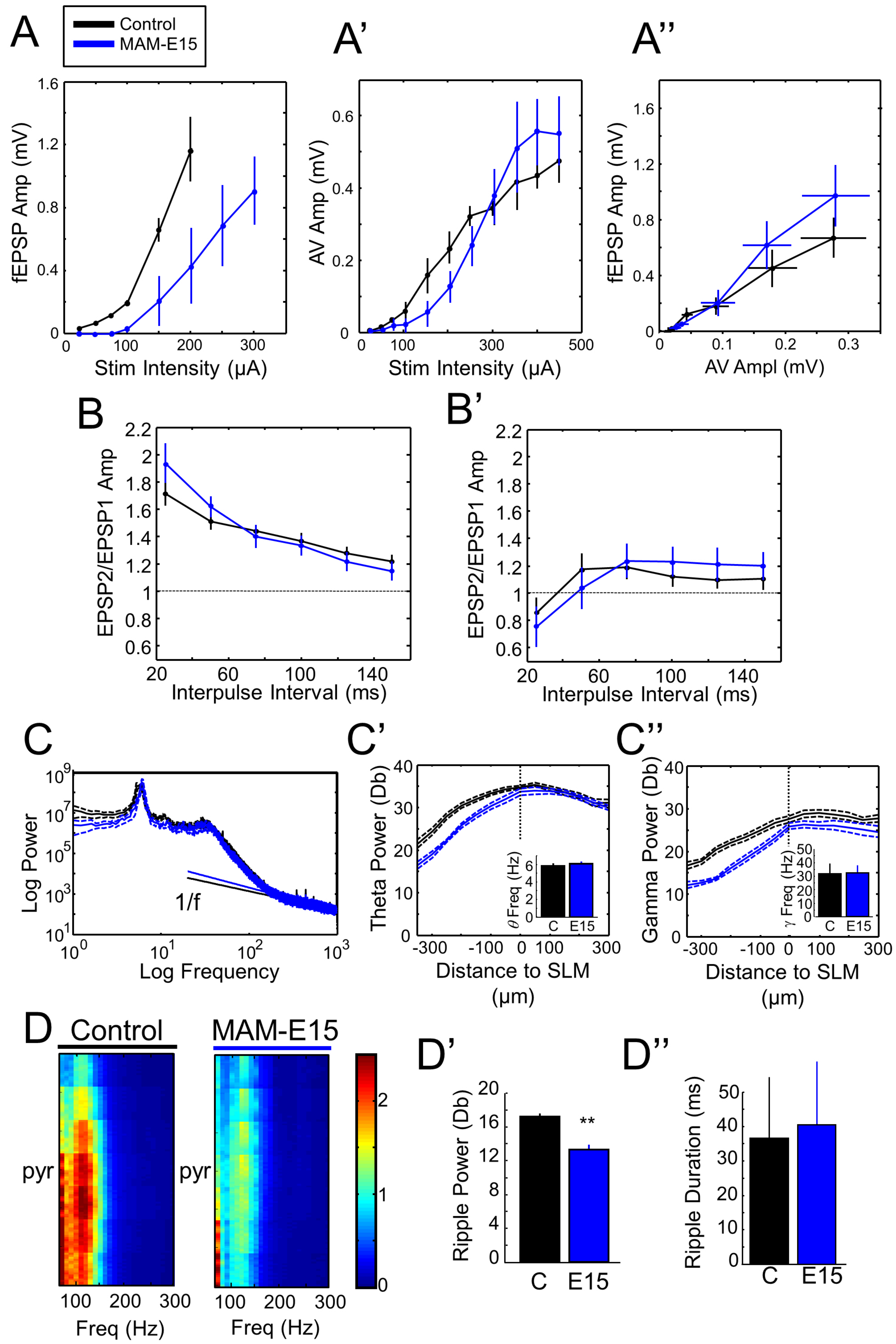

Supplement: Supplementary Figure 1 — Paired-pulse inhibition and facilitation. (A) (Left) Paired-pulse inhibition (using maximal stimulation intensities) of the second response at inter-pulse intervals of 25, 50, 75, 100, 125, and 150 ms was used to probe for inhibitory circuits in different groups of animals, including doublecortin knockouts (Dcx-KO). (Right), No differences were evident between groups. Data from n = 9 control (non-manipulated), n = 6 MAM and n = 7 Dcx-KO. Saline-injected mice were similar to control. (B) (Left) Paired-pulse facilitation (using stimulation intensities of 40% of the maximal response) of the second response was used to probe for differences on presynaptic release. (Right), No clear differences emerged between groups. Data from n = 7 control (non-manipulated), n = 8 MAM and n = 8 Dcx-KO. Saline-injected mice were similar to control. [file Presentation1.PDF]

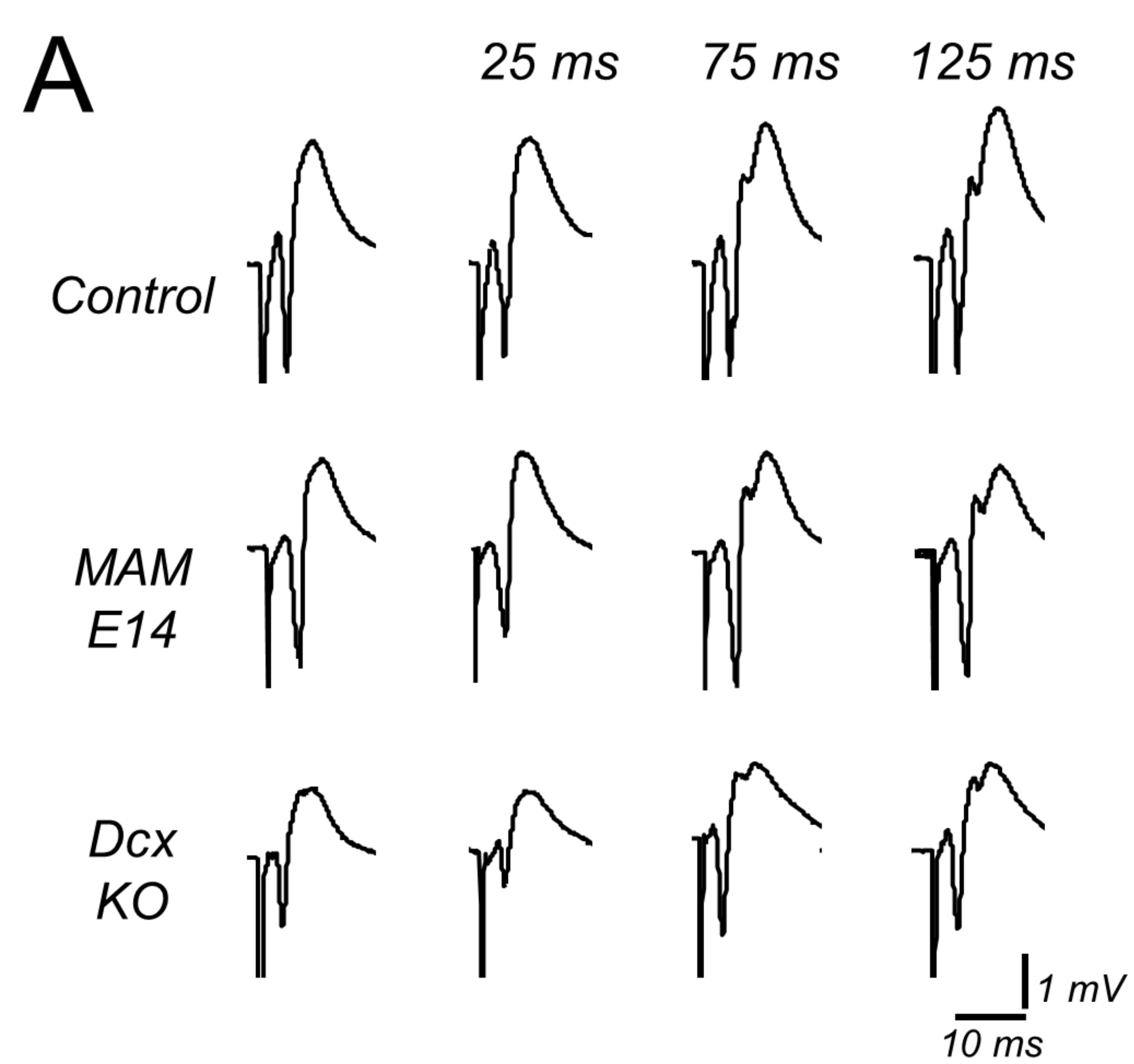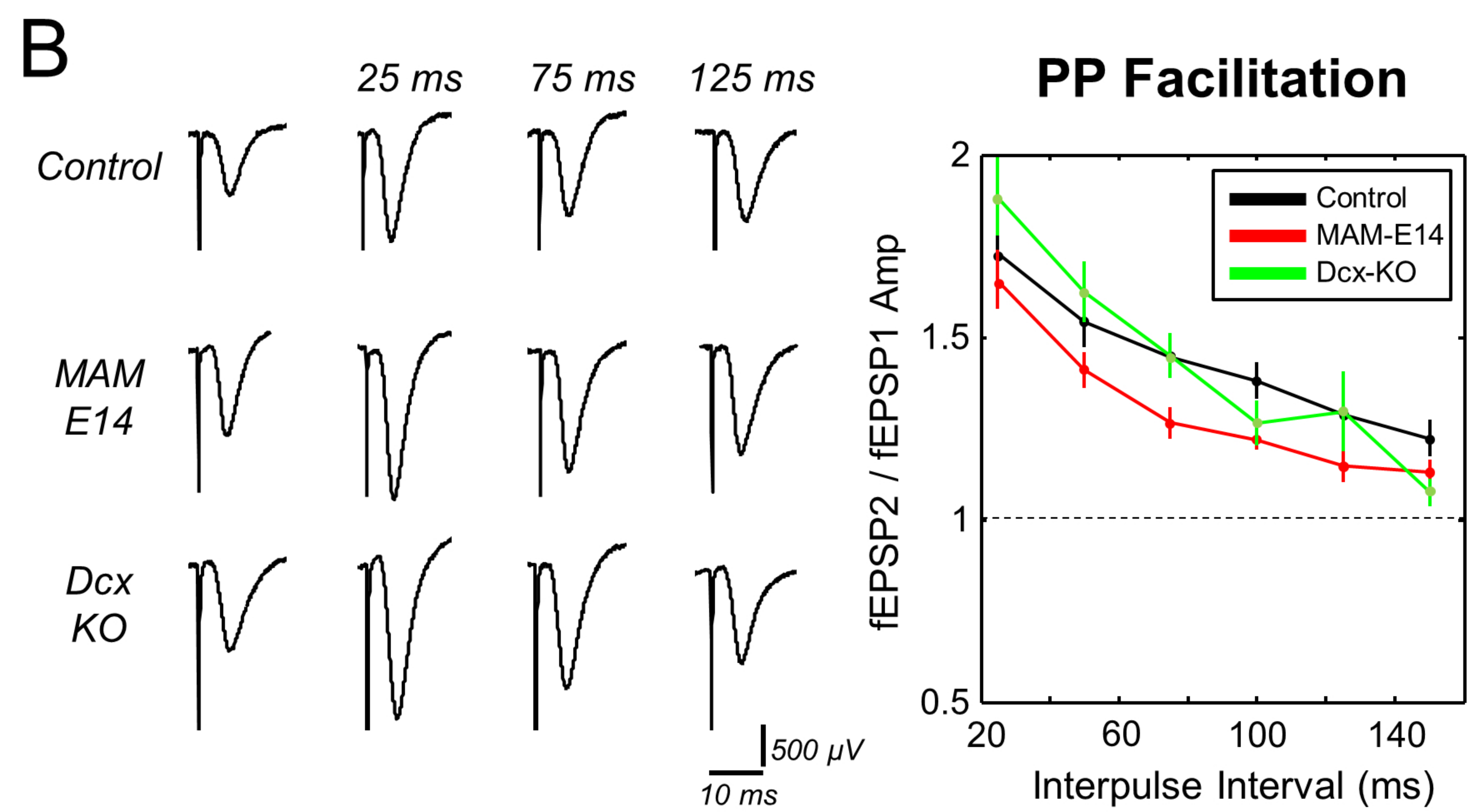

Supplement: Supplementary Figure 2 — Electrophysiological recordings in E15 MAM-injected mice under urethane. (A) Relationship between the amplitude of the fEPSP and stimulation intensity. Data from n = 11 control (non-manipulated), n = 5 MAM, and n = 7 Dcx-KO. (A′) Relationship between the amplitude of the afferent volley (AV) and stimulation intensity. (A″) Input/output curves suggesting slightly higher excitability in E15 MAM-treated mice as compared to control. Data in (A′,A″) are from n = 5 control (non-manipulated), n = 3 MAM, and n = 3 Dcx-KO. Saline-injected (sham) mice were similar to control. (B) Group data on paired-pulse facilitation (n = 7 control, n = 5 MAM, and n = 8 Dcx-KO). (B′) Group data on paired-pulse inhibition. (n = 9 control non-manipulated mice, n = 4 MAM, and n = 7 Dcx-KO). No differences were found between groups. Saline-injected (sham) mice were similar to control. (C) Mean power spectrum data from control (black, n = 10 non-manipulated mice) and E15 MAM-injected mice (blue, n = 5) as recorded at the slm electrode. Discontinuous line represents 95% confidence interval. Note deviation from 1/f noise in all cases, and dominant peaks at theta (4–6 Hz) and gamma (30–40 Hz) frequency. (C′) Spatial distribution of the normalized (1/f corrected) theta power area (4–10 Hz) around the stratum lacunosum moleculare (taken as reference). There is statistical difference between groups especially at CA1 [F(1, 7)= 32.03; p < 0.001]. Inset plots group data on theta frequency peak showed no difference at the stratum lacunosum moleculare. (C″) Spatial distribution of the gamma power area (30–90 Hz) around the slm channel (taken as reference) showed differences between groups [F(1, 7) = 44.87, p < 0.001]. The inset plots group differences of gamma frequency peak. (D) Potential differences of sharp-wave ripple events in control and E15 MAM-injected mice. High-frequency oscillations (ripples) at 100–150 Hz were maximal around the stratum pyramidale. (D′) Similar to E14 injected mice, ripple powe [file Presentation2.PDF]
